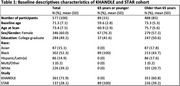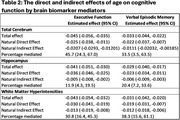# Neuroimaging biomarkers as a mediator of age and cognition in diverse cohorts

**DOI:** 10.1002/alz70856_105302

**Published:** 2026-01-07

**Authors:** Yi Lor, Alexander Ivan B. Posis, Batool M. Rizvi, Kristen M. George, Dan M. Mungas, Paola Gilsanz, Rachel A. Whitmer

**Affiliations:** ^1^ University of California, Davis, Davis, CA, USA; ^2^ University of California, Davis, Sacramento, CA, USA; ^3^ Kaiser Permanente Northern California Division of Research, Pleasanton, CA, USA

## Abstract

**Background:**

Age is associated with changes in brain integrity and cognition, but it is not agreed on whether age should be controlled for in statistical models. If age substantially contributes to brain integrity, then we may expect that brain integrity mediates the association of age and cognition. Therefore, we tested whether markers of brain integrity mediate the association of age and cognition.

**Method:**

Adults ages 50+ from two harmonized cohorts (Kaiser Healthy Aging and Diverse Life Experience, and Study of Healthy Aging in African Americans) who received a 3T brain MRI were included. Executive function (EF) and verbal episodic memory (VEM) were measured using a neuropsychological battery and z‐scored. Linear regression model was used to assess the association of baseline age and follow‐up cognition, adjusting for sex and education. Using mediation analyses, we decomposed the total effect of age into the natural direct and indirect effects via neuroimaging biomarkers of z‐standardized total cerebrum volumes, hippocampal volumes, and log‐white matter hyperintensities (WMH).

**Result:**

Among 577 participants (mean age=71.3; SD=7.1), 60% were females and 49% had ≥college education. The total effect of age on EF was (β=‐0.04; 95% CI ‐0.05, ‐0.03) and VEM was (β=‐0.02; 95% CI ‐0.04, ‐0.02). The direct effects of age on EF when not mediated is β(95% CI)=‐0.025(‐0.038, ‐0.011) via total cerebrum, β(95% CI)=‐0.036(‐0.046, ‐0.025) via hippocampus, and β(95% CI)=‐0.030(‐0.041, ‐0.019) via WMH. Similarly, the direct effect of age on VEM when not mediated is β(95% CI)=‐0.022(‐0.037, ‐0.007) via total cerebrum, β(95% CI)=‐0.023(‐0.034, ‐0.011) via hippocampus and β(95% CI)=‐0.019(‐0.031, ‐0.007) via WMH. In separate mediation analyses, the association of age on EF was 45.7% (95% CI=24.3, 67.0) mediated through total cerebrum, 11.9% (95% CI=4.3, 19.5) through the hippocampus, and 30.8% (95% CI=16.4, 45.3) through WMH. The association of age on VEM was mediated 33.5% (95% CI=3.5, 63.5) through total cerebrum, 20.4% (95% CI=7.2, 33.6) through hippocampus, and 38.3% (95% CI=15.6, 61.1) through WMH.

**Conclusion:**

Neuroimaging biomarkers partly mediate the effect of age on cognition. Brain integrity is associated with cognition; therefore, the effects of neuroimaging markers should be carefully considered when assessing the association of age on cognition.